# Integrative Signaling Networks of Membrane Guanylate Cyclases: Biochemistry and Physiology

**DOI:** 10.3389/fnmol.2016.00083

**Published:** 2016-09-15

**Authors:** Rameshwar K. Sharma, Teresa Duda, Clint L. Makino

**Affiliations:** ^1^The Unit of Regulatory and Molecular Biology, Research Divisions of Biochemistry and Molecular Biology, Salus UniversityElkins Park, PA, USA; ^2^Department of Physiology and Biophysics, Boston University School of MedicineBoston, MA, USA

**Keywords:** membrane guanylate cyclase, cyclic GMP, signal transduction, ANF-RGC, ROS-GC, ONE-GC, neuronal calcium sensor, bicarbonate

## Abstract

This monograph presents a historical perspective of cornerstone developments on the biochemistry and physiology of mammalian membrane guanylate cyclases (MGCs), highlighting contributions made by the authors and their collaborators. Upon resolution of early contentious studies, cyclic GMP emerged alongside cyclic AMP, as an important intracellular second messenger for hormonal signaling. However, the two signaling pathways differ in significant ways. In the cyclic AMP pathway, hormone binding to a G protein coupled receptor leads to stimulation or inhibition of an adenylate cyclase, whereas the cyclic GMP pathway dispenses with intermediaries; hormone binds to an MGC to affect its activity. Although the cyclic GMP pathway is direct, it is by no means simple. The modular design of the molecule incorporates regulation by ATP binding and phosphorylation. MGCs can form complexes with Ca^2+^-sensing subunits that either increase or decrease cyclic GMP synthesis, depending on subunit identity. In some systems, co-expression of two Ca^2+^ sensors, GCAP1 and S100B with ROS-GC1 confers bimodal signaling marked by increases in cyclic GMP synthesis when intracellular Ca^2+^ concentration rises or falls. Some MGCs monitor or are modulated by carbon dioxide via its conversion to bicarbonate. One MGC even functions as a thermosensor as well as a chemosensor; activity reaches a maximum with a mild drop in temperature. The complexity afforded by these multiple limbs of operation enables MGC networks to perform transductions traditionally reserved for G protein coupled receptors and Transient Receptor Potential (TRP) ion channels and to serve a diverse array of functions, including control over cardiac vasculature, smooth muscle relaxation, blood pressure regulation, cellular growth, sensory transductions, neural plasticity and memory.

## Introduction

Initially relegated to the shadows of cyclic AMP signaling, cyclic GMP has slowly ascended to eminence as an important independent signal in the body, governed by the activities of soluble and membrane guanylate cyclases (MGCs) and phosphodiesterases. Here, we briefly outline the chronological development of the field of mammalian MGCs, focusing on how the modular design of the receptor enzyme adds layers of complexity to its biochemistry and physiological function. Variations in the modules confer flexibility and enable MGCs to command a pervasive signaling role over diverse functions throughout the body. Some of the advancements made by the authors and their collaborators are featured. For more comprehensive coverage, the reader is referred to the authors’ earlier reviews: (Sharma (1978, 1985, 2002, 2010); Pugh et al. (1997); Sharma et al. (2004, 2014); Duda et al. (2005b, 2014); Sharma and Duda (2012); Sharma and Duda (2014a,b)).

### Cyclic GMP Is a Hormonal Second Messenger

An observation made five decades ago, that cyclic GMP exists in rat urine (Ashman et al., 1963) and the finding that its level in urine depends upon hormonal state (Hardman and Sutherland, 1969; Hardman et al., 1969) were early clues that cyclic GMP serves as a hormonal second messenger. Guided by a prototypic cyclic AMP template, this concept implicated a membrane receptor guanylate cyclase transduction system in vertebrates. In the beginning, there was passionate enthusiasm for this concept and in support, cyclic GMP was present and guanylate cyclase catalytic activity was detected in all tested tissues (Goldberg et al., 1969, 1973; Ishikawa et al., 1969). But with reports that guanylate cyclase catalytic activity was stimulated by a variety of non-hormonal ligands: polyunsaturated fatty acids, peroxides, hydroperoxides, free radicals, ascorbic acid, sodium nitroprusside, and cigarette smoke (Goldberg and Haddox, 1977; Murad et al., 1979), a notion took hold in which MGC was a non-specific enzyme regulated by the cell’s oxidation-reduction potential. In extreme views, no component of cyclic GMP turnover was regarded as linked directly to an intracellular signaling pathway. Where it was found, cyclic GMP turnover was believed to be subordinate to the cyclic AMP signaling system. As a case in point, the known cyclic GMP-dependent protein kinase activity showed some cross-reactivity with cyclic AMP and was thus interpreted as a means for augmenting cyclic AMP signaling (Gill and McCune, 1979).

A few groups pushed on, not willing to relinquish a primary role for cyclic GMP as a second messenger role in hormonal signal transduction (reviewed in Sharma, 1978, 1985; Sharma et al., 1988a,b; Pugh et al., 1997; Sharma and Duda, 1997, 2012, 2014a,b; Sharma, 2002, 2010; Duda et al., 2005b, 2014; Sharma et al., 2015). Two key observations proved to be instrumental in resolving the matter. First, an ACTH-sensitive MGC transduction system exists in rat adrenocortical carcinoma 494 cells and in isolated adrenal fasciculata cells (Perchellet and Sharma, 1980; Shanker and Sharma, 1980; Jaiswal and Sharma, 1986; Jaiswal et al., 1986; Venkataraman et al., 1998; reviewed in Sharma, 2010). Second, unlike adrenal homogenates, isolated adrenal fasciculate and adrenocortical carcinoma cells lack cyclic AMP phosphodiesterase activity. These systems were therefore ideal for testing whether cyclic AMP is the sole hormonal second messenger or whether ACTH evokes MGC activity (Kitabchi et al., 1971; Sharma, 1972).

**Figure 1:**
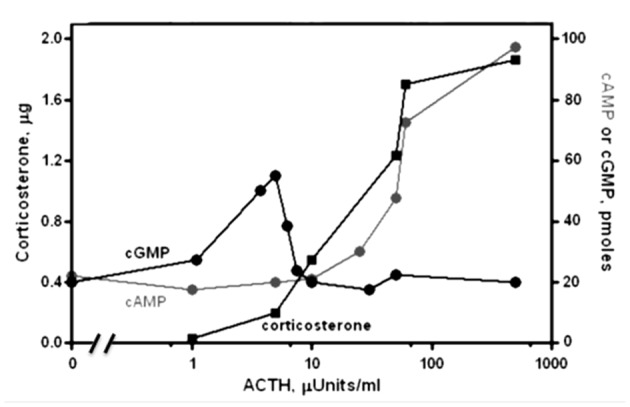
**Dose-response relations for the levels of cyclic GMP, cyclic AMP and corticosterone as a function of ACTH in suspensions of isolated adrenal cells of rat.** Cyclic GMP (black circles) and corticosterone (black squares) are produced at [ACTH] too low to affect cyclic AMP synthesis. At higher [ACTH], activation of a phosphodiesterase counteracts GMP synthesis and cyclic AMP levels (gray circles) rise to further increase steroidogenesis. Modified, with permission, from Sharma et al. (1974). One micro unit (μU) of ACTH is equal to 4.5 pg or 1.2 × 10^−13^ moles.

Physiological levels of ACTH (1–10 μU) stimulate steroidogenesis, yet do not raise the level of cyclic AMP (Figure 1; Sharma et al., 1974). Moreover, there exists an excellent temporal relation between cyclic GMP formation (Sharma et al., 1974; Harrington et al., 1978), phosphorylation and steroidogenesis (Sharma, 1973; Sharma et al., 1976; Perchellet et al., 1978) at these hormone concentrations. Exogenous cyclic GMP mimics ACTH in stimulating protein kinase activity, followed by conversion of cholesterol to corticosterone (Sharma et al., 1976; Sharma and Sawhney, 1978). Eventually, direct evidence was obtained for the presence of ACTH/Ca^2+^-dependent MGC activity in adrenocortical and adrenocortical carcinoma plasma membranes (Nambi and Sharma, 1981a,b; Nambi et al., 1982a,b). At levels an order of magnitude higher, ACTH will elevate cyclic AMP and drive steroidogenesis further but these levels extend into the supra-physiological range (Sharma et al., 1974, 1976). Thus, cyclic GMP is the principal physiological second messenger of ACTH in isolated adrenal fasciculate and adrenocortical carcinoma cells and cyclic AMP is not the sole second messenger for hormonal signaling in the body.

Two challenges were mounted against these ideas. First, a dibutyryl cyclic AMP analog was the most effective activator of cyclic AMP-dependent protein kinase, yet dibutyryl cyclic GMP evoked far less steroidogenic activity than its parent compound cyclic GMP (Hayashi et al., 1979). Second, incubation of adrenocortical cells with sodium nitroprusside and ascorbic acid incremented cyclic GMP levels without stimulating the production of corticosterone (Laychock and Hardman, 1978). Both challenges were overcome upon purification and characterization of the cyclic GMP-dependent protein kinase from the adrenal cortex (Ahrens et al., 1982). In a playful twist of NATURE, the cyclic GMP-dependent protein kinase differs from the cyclic AMP-dependent protein kinase in several curious ways. The most potent effector molecule for cyclic GMP-dependent protein kinase is 8-bromo cyclic GMP; dibutyryl cyclic GMP is almost totally ineffective (Ahrens et al., 1982). Perhaps more surprisingly, it turns out that nitroprusside and ascorbate inhibit cyclic GMP-dependent protein kinase activity (Sharma et al., 1988a,b).

After a heme-dependent soluble guanylate cyclase was purified from bovine lung (Ignarro et al., 1982a,b), it became clear that only the soluble guanylate cyclase is stimulated by free radical-, nitric oxide (NO)-generating agents, including heme; such agents produce little or no effect on MGC (Nambi et al., 1982b; Sharma et al., 1989b). It was thus established that guanylate cyclases exist in membrane and soluble forms each with distinct biochemical properties, and that their signaling roles do not necessarily overlap. In rat adrenal cortex, most (80%) of the guanylate cyclase is membrane-bound and only a minor amount (20%) is soluble (Nambi et al., 1982b; Sharma et al., 1989b). In the second challenge above, it was this minor fraction that was stimulated by sodium nitroprusside and ascorbic acid.

**Figure 2:**
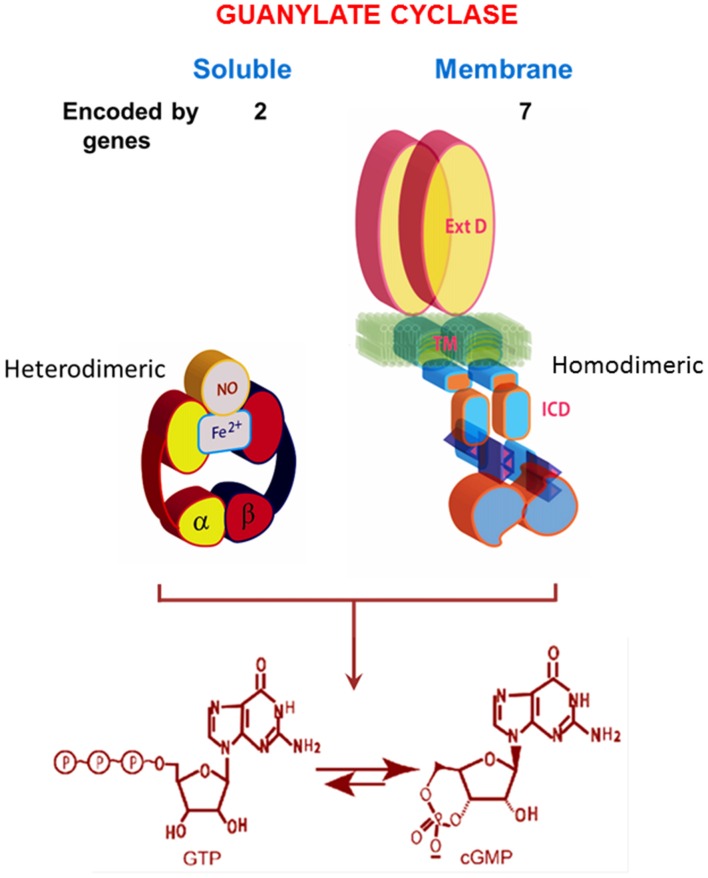
**Comparison of soluble and membrane guanylate cyclases (MGCs).** The soluble form is a heterodimer of α and β subunits with a single heme capable of binding nitric oxide (NO). The C-terminal segments of both subunits contribute to the catalytic center. The MGC is a single transmembrane spanning protein that is active as a homodimer. Heme binding does not occur. A transmembrane domain (TM) divides the protein into two roughly equal parts, an extracellular domain (ExtD) and an intracellular domain (ICD). Within the ICD, corresponding C-terminal segments from each monomer, arranged in an antiparallel orientation, form the catalytic center. Both types of cyclases are lyases (EC4.6.1.2) that catalyze synthesis of cyclic GMP from GTP. Note that the relative sizes of the modules are not drawn to scale. Redrawn, with permission, from Sharma and Duda (2014a).

In its current status, the soluble form of the guanylate cyclase is encoded by two genes [the functionalities of β2 cloned from rat kidney and α2, from human fetal brain, remain to be determined (Denninger and Marletta, 1999)]. It is heterodimeric and requires heme for its activity (Figure 2; Allerston et al., 2013; Sharma and Duda, 2014a). As will be described below, MGCs are quite different. In mammals, MGCs are encoded by seven different genes and although dimerization is requisite for activity, it would appear that only homodimers are formed *in situ*.

### Atrial Natriuretic Factor Receptor Guanylate Cyclase (ANF-RGC) Was the First Membrane Guanylate Cyclase to be Purified and Characterized

Purification and characterization of an MGC from rat adrenocortical carcinoma (Paul, 1986; Paul et al., 1987; Sharma, 1988; Sharma and Duda, 2014b) and subsequently from adrenal cortex (Takayanagi et al., 1987; Meloche et al., 1988) brought the discovery that it is a receptor for atrial natriuretic factor (ANF). This receptor MGC, named ANF-RGC, provides the basis for ANF-stimulated cyclic GMP and corticosterone synthesis (Jaiswal et al., 1986). Presumably a similar MGC responds to ACTH, but confirmation is not yet available. The original report noted, “coexistence of the ANF receptor and guanylate cyclase activities on a single polypeptide chain indicates that the mechanism of transmembrane signal transduction involving mediation by second messenger, cyclic GMP, is different from the well-established adenylate cyclase system. In hormone-dependent adenylate cyclase there is an assemblage of individual components—receptor, GTP binding protein, and catalytic moiety—for signal transduction. In contrast, the presence of dual activities—receptor binding and enzymatic—on a single polypeptide chain indicates that this transmembrane protein contains both the information for signal recognition and its translation into a second messenger” (Paul et al., 1987). Accordingly, a multimodal ANF-RGC signal transduction model was envisioned (Sharma et al., 1988a,b, 1989a) and refined (Sharma, 2002; Figure 2). Its central features were that a hormone receptor domain protruded into the extracellular space, a catalytic domain resided inside the cell and a transmembrane segment linked the two.

The same antibody used to purify ANF-RGC to homogeneity from adrenocortical carcinoma and from adrenal cortex indicated a much wider expression including testes (Marala and Sharma, 1988), neurons located in the ventral horn region of rat spinal cord, cerebellar Purkinje cells, and renal glomerular cells (Ballermann et al., 1988). The milestone discovery of ANF-RGC would revolutionize the basic cellular signal transduction field as well as the field of clinical cardiovascular physiology.

An intriguing finding of the early studies was the obligatory presence of Ca^2+^ for hormonal stimulation of steroidogenesis (Sayers et al., 1972; Haksar and Péron, 1973; Bowyer and Kitabchi, 1974), yet by itself, Ca^2+^ was ineffective. Only when present with the hormone, did it become an effective steroidogenic factor (Perchellet and Sharma, 1979). Although the underlying mechanism could have been indirect and has still not been clarified, Ca^2+^ would be found to play a profound role in regulating MGC activity and diversifying its functionality as the field of MGCs progressed.

### MGCs Are Constructed with a Modular Design

About 3-years post-purification, the mRNA of ANF-RGC was cloned (Chinkers et al., 1989; Lowe et al., 1989; Pandey and Singh, 1990; Duda et al., 1991). Cloning and sequencing of two more family members soon followed (Chang et al., 1989; Schulz et al., 1989; Duda et al., 1993c). Like ANF-RGC (or GC-A), the new guanylate cyclases are receptors for hormones: type C-natriuretic peptide for one receptor and guanylin, uroguanylin and heat stable enterotoxin for the other, hence, they were named CNP-RGC (or GC-B) and STa-RGC (or GC-C), respectively.

Hydropathy analysis suggests that all three receptors are modular, with nearly identical topographies. The extracellular portion forms the hormone-binding domain and embodies the maximal structural diversity. The intracellular portion starts with a kinase-like domain that stretches up to a catalytic domain, (Duda et al., 1991). By applying recombinant tools to MGCs and guided by the theoretical ANF-RGC structural template it was possible to test the multi-modularity concept, map the domains and define their functional components and predict the transmembrane migration of the hormonal signal at a sub-molecular level.

The first task was to establish that the extracellular domain (ExtD) of ANF-RGC truly does constitute the hormone’s (ANF) signaling site. A genetic variant of ANF-RGC (GCα), cloned from the adrenal cortex, has a structure that varies in just two amino acid residues from the ExtD of the true ANF-RGC: His^338^ substitutes for Gln and Pro^364^ substitutes for Leu (Duda et al., 1991). GCα supports normal basal catalytic activity, yet it is not an ANF receptor. Transformation of its residues 338 and 364 to the true ANF-RGC residues confers ANF-binding and regulatory activities indicating the necessity for Gln^338^ and/or Leu^364^. Subsequent point mutation analyses single out Leu^364^ as the key residue for controlling both activities. Historically, GCα was the first naturally occurring ANF-RGC mutation functionally linked with hormonally-dependent core catalytic domain (CCD) activity (Duda et al., 1991).

The amino acid residues in CNP-RGC corresponding to Gln^338^ and Leu^364^ in ANF-RGC are Glu^332^ and Val^358^, respectively. For CNP-RGC, Glu^332^ in the ExtD is critical for C-type natriuretic peptide (CNP) binding and CNP-dependent catalytic regulatory activity (Duda et al., 1993c, 1994). As with Leu^364^ in ANF-RGC, the site has no influence on the basal catalytic activity of the guanylate cyclase. The folding patterns of the external domains of these two guanylate cyclases are nearly identical (Duda et al., 1994). Substitution of the Gln^338^ residue with Glu endows ANF-RGC with significant CNP signal transduction activity (Duda et al., 1995). Conversely, replacing Val^358^ with Leu generates significant ANF signal transduction activity in CNP-RGC. These two residues therefore perform equivalent functions in controlling the ligand specificities of ANF-RGC and CNP-RGC. Subsequent X-ray crystallographic analyses provided validation and revealed the ExtDs to exist as homo-dimers in a head to head conformation (He et al., 2001; Ogawa et al., 2004, 2009).

### Hormonal Signal Transduction Is ATP-Regulated

An enigmatic observation was that despite binding ANF stoichiometrically, the catalytic activity of purified ANF-RGC was not stimulated (Paul et al., 1987). Two groups solved this puzzle (Chinkers et al., 1991; Marala et al., 1991); ATP is obligatory for ANF-dependent ANF-RGC activity. Neither ANF nor ATP alone is sufficient. Since two nonhydrolyzable analogs, ATPγS and AMP-PNP, mimic ATP with EC_50_s between 0.3–0.5 mM (Duda et al., 1993a), phosphorylation need not occur and ATP must be an allosteric regulator (Chinkers et al., 1991; Marala et al., 1991). With Hill coefficients near 1 (Duda et al., 2011b), it is not clear whether one ATP per dimer is adequate or whether two ATPs drive greater activity than one. Importantly, the identical situation exists for CNP transduction by recombinant CNP-RGC (Duda et al., 1993c).

Definition of the mechanism by which ATP controls transmission of the hormonal signal at the sub-molecular level was achieved through two decades of programmed study (reviewed in Sharma, 2010). A vaguely termed, “kinase homology domain (KHD)” of ANF-RGC extends from a juxtamembrane domain (JMD) to the CCD (Chinkers and Garbers, 1989; Marala et al., 1992). Within the KHD, a glycine rich cluster (GRC), Gly^503^-Arg-Gly-Ser-Asn-Tyr-Gly^509^ bestows ATP-dependence to ANF transduction (Goraczniak et al., 1992). Hence, this motif was more aptly renamed ATP-regulatory module (ARM). The ARM in CNP-RGC is Leu^497^-Arg-Gly^499^-Ser-Ser-Tyr-Gly^503^. If the ANF-RGC-ARM sequence is changed to its CNP-RGC counterpart, the ATP dependence of the ANF response is preserved (Duda et al., 1993a). Together with the earlier finding that the entire KHDs of ANF-RGC and CNP-RGC are interchangeable (Koller et al., 1992), the transduction mechanisms of these two MGCs appear to function identically. Evaluation of individual glycine residues in the ARM sequence homed in on Gly^505^ in ANF-RGC and the identically placed Gly^499^ in CNP-RGC as critical for ATP binding and ANF signal transduction (Duda et al., 1993b).

**Figure 3:**
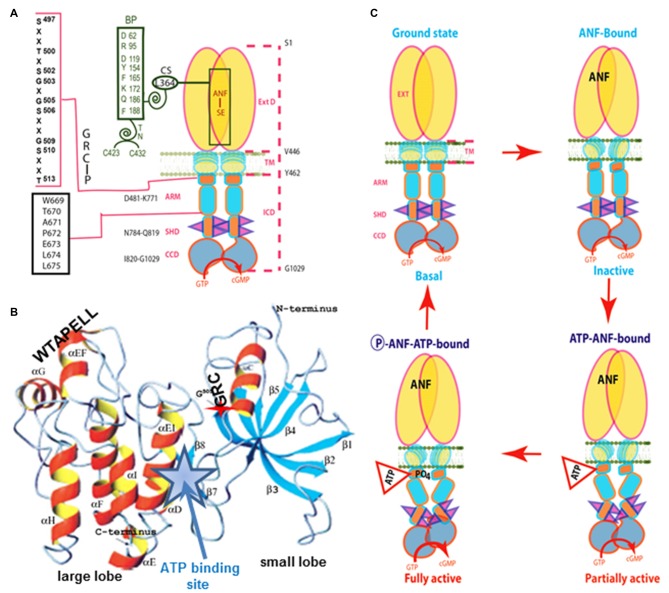
**Model for signal transduction by atrial natriuretic factor-receptor guanylate cyclase (ANF-RGC). (A)** Important module segments involved in activation. An ANF-signaling element (ANF-SE) resides in the ExtD. A Central Switch (CS), L364, controls the ANF binding site. A Binding Pocket (BP) is hinged with the CS (van den Akker et al., 2000; Ogawa et al., 2004, 2009). Two disulfide bridged cysteine residues act as a Transduction Node (TN) to guide the transmembrane migration of the ANF signal to an intracellular, ATP-Regulated Module (ARM; Duda et al., 2005b). ATP amplifies the ANF signal by bringing two critical domains to the surface: a glycine rich cluster (GRC), G-X-G^505^-X-X-X-G, making surrounding serine and threonine available for phosphorylation (GRC-P) and a 7-aa residue W^669^-TAPELL^675^ motif to activate the core catalytic domain (CCD). **(B)** Structure of the ARM in its apo form. Four antiparallel β strands and one helix constitute the small lobe. The large lobe is made up of eight α helices and two β strands. The positions of the key G^505^ residue of the GRC motif within the small lobe and of the W^669^-TAPELL^675^ motif within the large lobe are indicated. ATP binding is sandwiched between the two lobes (indicated by a star; modified, with permission, from Duda et al., 2000; Sharma et al., 2001). **(C)** Activation model for ANF-RGC. Binding of an ANF molecule to the ExtDs of the dimer primes the ANF-SE, by rotating TN. The twisting motion propagates through TM to prepare ARM for ATP binding (Ogawa et al., 2004; Parat et al., 2010). ATP binding triggers a cascade of temporal and spatial changes (Duda et al., 2001c). With G^505^ in GRC-P acting as a pivot, the ATP BP shifts its position and its floor rotates. There is movement of ARM’s β4 and β5 strands and the loop between them and movement of the αE and F helices that exposes the hydrophobic WTAPELL motif for interaction with CCD (Duda et al., 2009). These structural rearrangements initiate 50% maximal catalytic activity. Full activation is attained after multiple serines and threonines in GRC-P become phosphorylated (Duda et al., 2011b). The conformational changes wrought by ATP binding reduce the affinity of ANF-RGC for ANF and phosphorylation lowers the affinity for ATP binding. Dissociation of ANF and ATP return ANF-RGC to its ground state. Modified with permission, from Duda et al. (2011b).

The ANF-RGC ARM domain was simulated through homology-based modeling, patterning it after the x-ray crystal structures of insulin receptor kinase and hematopoietic cell kinase. In turn, its functional elements were experimentally decoded through point mutation/expression, time-resolved tryptophan fluorescence and Forster Resonance Energy Transfer, reconstitution and mass spectroscopic studies (Figure 3A). The greater ARM boundaries stretch from residues 481–771. A smaller, 91 residue N-terminal lobe (496–586) connects to a larger, 185 residue C-terminal lobe (587–771; Duda et al., 2000; Sharma et al., 2001; Figure 3B). ATP binding partially amplifies the ANF signal by repositioning a W^669^-TAPELL^675^ motif in the larger lobe. This repositioning is critical for hormone-dependent activation of ANF-RGC; deletion of the WTAPELL motif results in an ANF-RGC that is unresponsive to ANF and ATP in the recombinant system (Duda et al., 2009) and in a genetically modified mouse model, causes hypertension and cardiac hypertrophy (Duda et al., 2013). Thus, the seven-residue encoded motif of ANF-RGC gene exerts total control over the hormonal regulation of blood pressure.

Binding of ATP also causes buried serine and threonine residues in GRC-P within the smaller lobe of the ARM to shift to the surface, whereupon they are phosphorylated by a hypothetical kinase. Full amplification of the ANF signal is achieved by phosphorylation. Maximal activity is self-limiting, however, because ANF-RGC loses affinity for ANF upon ATP binding (Larose et al., 1991; Jewett et al., 1993; Duda and Sharma, 1995a,b) and loses affinity for ATP after phosphorylation.

Based on additional observations that dephosphorylation of ANF-RGC underlies homologous desensitization (Potter and Garbers, 1992), we propose a working model for activation (Figure 3C). ATP and ANF can bind ANF-RGC independently, but in the absence of ATP, ANF cannot stimulate cyclic GMP synthesis. ATP binding exposes six serines and threonines in the ARM, enabling them to be phosphorylated. Dephosphorylation can occur but the residues can then be re-phosphorylated. The conformational changes also lower the affinity for ANF binding. Phosphorylation promotes ATP release, which helps return of the receptor to the basal state by reducing activity and slowing the rate of phosphorylation. Dephosphorylation continues as long as ANF is bound. When ANF dissociates, the serines and threonines in ARM fold back into the receptor and lose access to phosphatase and kinase. If dephosphorylation is complete, then the receptor can bind ANF, but is completely inactive until it also binds ATP and is re-phosphorylated. But with residual phosphorylation, ANF binding alone elicits weak activity. Full activation requires ATP binding and phosphorylation of all residues. A central theme of the model is that it is dynamic. Notably, this operational mode selectively controls the regulatory activity of ANF without affecting the basal catalytic activity of ANF-RGC. It appears that the same model applies to the ligand-dependent activation of CNP-RGC and STa-RGC (Potter and Hunter, 1998; Bhandari et al., 2001; Jaleel et al., 2006).

### MGCs Transduce Intracellular Ca^2+^ Signals

Phototransduction, the conversion of light into an electrical signal in the outer segments of rod and cone photoreceptors, marks the first step in the visual process. By the 1980s, it was known that both Ca^2+^ and cyclic GMP were critical cytosolic regulators of the photon response. Eventually, cyclic GMP was proven to be the second messenger of phototransduction when it was shown to increase directly the conductance of a new class of ion channels for which phosphorylation was not necessary (Fesenko et al., 1985). This discovery brought a major paradigm shift because until that time, cyclic nucleotides were thought to act exclusively through a kinase intermediary. However, critical issues remained: What was the source of the cyclic GMP? How did Ca^2+^ fit in? (reviewed in Pugh and Cobbs, 1986; Stryer, 1986).

To find answers, a search for the molecular identity of the photoreceptor ROS-GC was undertaken. After initial failures (Horio and Murad, 1991a,b; Shyjan et al., 1992), in a cutting edge finding, a protein was successfully purified from bovine rod outer segments (ROS) and its sequence was pieced together from protein fragments. Subsequent sequence-based molecular cloning established the true molecular structure of ROS-GC1 (Margulis et al., 1993; Goraczniak et al., 1994). Based on the bovine ROS-GC1 structure, the molecular structure of the original ret-GC (Shyjan et al., 1992) was corrected in 1995 to show that it is the human version of the bovine form (Lowe DG, accession number M92432). Notably, ROS-GC1 is the lone MGC to have its molecular identity established on the basis of protein sequence. Comparison of the results to those obtained by cloning led to the realization that ROS-GC is preceded by an N-terminal leader sequence (LS) that gets deleted post-translationally. The theoretical molecular mass of the protein with the 56-amino acid LS is 120, 361 Da; without it, it is 114, 360 Da (Goraczniak et al., 1994). The 114, 360 Da molecular mass was similar to the previously biochemically characterized bovine (Koch, 1991) and toad photoreceptor guanylate cyclases (Hayashi and Yamazaki, 1991). Unlike ANF-RGC and CNP-RGC (Paul et al., 1987; Chang et al., 1989; Chinkers et al., 1989; Duda et al., 1993c), ROS-GC1 is not modulated by natriuretic peptide hormone (Goraczniak et al., 1994). For that matter, no extracellular ligands have ever been identified, so ROS-GC1 remains an orphan surface receptor. A second ROS-GC (ROS-GC2) was discovered in bovine retina (Goraczniak et al., 1997), the human version was termed Ret-GC2 (Lowe et al., 1995). Recombinant ROS-GC1 and ROS-GC2 organize into homodimers and heterodimers, but few if any heterodimers form in bovine retina despite the co-expression of both guanylate cyclases in rods (Yang and Garbers, 1997).

**Figure 4:**
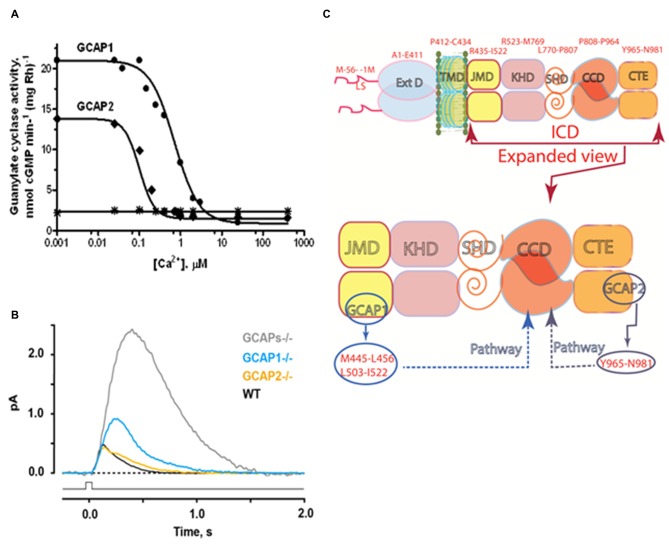
**Ca^2+^- sensing subunits of rod outer segment-guanylate cyclase (ROS-GC). (A)** Ca^2+^-dependence of ROS-GC1 activity in washed bovine ROS membranes. In the presence of recombinant guanylate cyclase activating protein (GCAP), activity is stimulated when Ca^2+^ is low and suppressed when Ca^2+^ is high. The “IC_50_” for Ca^2+^ was 707 ± 122 nM for GCAP1 and 100 ± 32 nM for GCAP2. The dose response curves were cooperative with Hill coefficients of 1.5 and 2.4 for GCAP1 and GCAP2, respectively. Results from Hwang et al. (2003). The difference in IC_50_ values for Ca^2+^ between GCAP1 and GCAP2, observed for the first time in this study, inspired the proposal of the “Ca^2+^ relay” model for the GCAP1 and GCAP2 modulation of ROS-GC activity (Koch and Dell’Orco, 2013). **(B)** Effects of GCAPs on the photon responses of rods from WT, GCAP1^−/−^, GCAP2^−/−^ and GCAPs double knockout (GCAPs^−/−^) mice. In the absence of both GCAPs, the single photon response rises for a longer period to an amplitude ~5 fold larger than normal and recovers very slowly. Knockout of GCAP1 alone results in a compensatory overexpression of GCAP2. The rising phase of the photon response is not quite as prolonged as with GCAPs^−/−^ but the amplitude is still ~2 fold larger than normal. GCAP1 levels appear to be normal after knockout of GCAP2. The photon response is normal in amplitude but takes somewhat longer to recover (Results from Wen et al., 2014). **(C)** Modular construction of the ROS-GC1 dimer. A 56 amino acid leader sequence (LS) precedes the ExtD in the nascent, immature protein. All signaling events occur in ICD, which is composed of: JMD, juxtamembrane domain; KHD, kinase homology domain; SHD, signaling helix domain; CCD, catalytic core domain; and CTE, C-terminal extension. Two specific switches for Ca^2+^ sensing subunits, one for GCAP1 in the JMD, and one for GCAP2 in the CTE, are located on opposing sides of the CCD. The MGC complex exists as a dimer of homodimers in which two ROS-GC1s combine either two GCAP1s or two GCAP2s.

In a groundbreaking discovery, a soluble bovine ROS fraction stimulated the catalytic activity of photoreceptor guanylate cyclase in the absence of Ca^2+^ (Koch and Stryer, 1988). Three separate groups purified two similar but molecularly distinct guanylate cyclase activating proteins (GCAPs): GCAP1 (Palczewski et al., 1994; Subbaraya et al., 1994; Gorczyca et al., 1995; Frins et al., 1996) and GCAP2 (Dizhoor et al., 1995). For ROS-GC1 expressed in a heterologous system of COS cells catalytic activity increases 4–5 fold with the addition of GCAP1 at 10 nM Ca^2+^. Activity then falls approximately 6-fold as Ca^2+^ is raised to 1 μM with an IC_50_ near 100 nM (Duda et al., 1996b; Figure 4A). The small discrepancy between these magnitudes of activity change comes about because GCAP becomes an inhibitor at high Ca^2+^ (Dizhoor and Hurley, 1996). ROS-GC1 in membranes of the photoreceptor outer segments membranes treated with exogenous GCAP1 or GCAP2 behaves similarly (Figure 4A). These results along with cross-linking experiments (Duda et al., 1996b) affirm the association of GCAP1 with ROS-GC1 at low and high Ca^2+^ levels, making GCAP1 a Ca^2+^-sensing subunit of the cyclase.

Recombinant GCAP2 provides more powerful stimulation of recombinant ROS-GC1 at low Ca^2+^, but the EC_50_ of ROS-GC1 for GCAP2 of 6–8 μM is nearly an order of magnitude higher than the EC_50_ of ~0.8 μM for GCAP1 (Duda et al., 1996b; Goraczniak et al., 1998; Krishnan et al., 1998). ROS-GC2 is stimulated by GCAP2 at low Ca^2+^ by 12-fold with an EC_50_ of 1 μM. However, GCAP1 is not capable of stimulating ROS-GC2 or ANF-RGC at low Ca^2+^ (Duda et al., 1996b; Goraczniak et al., 1998). The binding sites on ROS-GC1 for the two Ca^2+^ sensing subunits were mapped by peptide competition and mutagenesis experiments (Figure 4C). Two short sequences, M^445^-L^456^ and L^503^-I^522^, in the JMD of ROS-GC1 are critical and specific for GCAP1 activation (Lange et al., 1999). The regulatory site for GCAP2, Y^965^-N^981^, is located in a C-terminal extension (CTE) of the CCD (Duda et al., 2005a). Despite their having binding sites positioned on opposite sides of the CCD, both GCAPs utilize the W^657^-TAPELL^663^ motif in ARM to amplify their stimulation of catalytic activity at low Ca^2+^ (Duda et al., 2011a). ATP binding is not obligatory, but will raise basal catalytic activity and GCAP1-stimulated activity at low Ca^2+^ by the same increment (Gorczyca et al., 1994; Aparicio and Applebury, 1996). Remarkably, ROS-GC exhibits intrinsic kinase activity and autophosphorylates four serine residues in its ARM (Aparicio and Applebury, 1996; Bereta et al., 2010). At present, the purpose of ROS-GC1 phosphorylation is a complete mystery; it produces no change in basal or GCAP1-stimulated activities and its occurrence is unrelated to Ca^2+^ regulation by GCAP1.

Rods and cones are not responsive to light in ROS-GC1/ROS-GC2 double knockout mice (Baehr et al., 2007) and guanylate cyclase activity loses its Ca^2+^ dependence in GCAP1/GCAP2 double knockout mice (Mendez et al., 2001). Expression of a third guanylate cyclase and a third GCAP linked with phototransduction is thereby excluded in mouse. In human retina there is a third GCAP capable of stimulating ROS-GC1 and ROS-GC2. It has Ca^2+^ regulatory properties similar to those of GCAP1 so its role in phototransduction is unclear (Haeseleer et al., 1999). Apparently mammalian photoreceptors utilize a Ca^2+^-modulated system composed of a pair of ROS-GCs and up to three GCAPs in their outer segments. Studies on mice deficient for either GCAP1 or GCAP2 (Makino et al., 2008, 2012) verify a relay model for how the two GCAPs operate in the rod (Koch and Dell’Orco, 2013). GCAP1 has the lower affinity for Ca^2+^ so it is the first responder to the light-induced fall in intracellular Ca^2+^. Over time after light onset and with higher intensities, the effect of GCAP1 saturates and stimulation of ROS-GC activity by GCAP2 becomes increasingly important. Together, the two GCAPs limit the growth of the photon response and accelerate the kinetics of the response recovery (Figure 4B). More complete accounts of the molecular properties of ROS-GCs and how ROS-GC systems control phototransduction may be found in Sharma and Duda (2014a); Wen et al. (2014); Koch and Dell’Orco (2015).

The discovery that intracellular Ca^2+^ regulates cyclic GMP synthesis in photoreceptor membranes shattered the concept that MGCs limit themselves to the transduction of hormones and other extracellular signals. Moreover it foreshadowed a greater complexity in signaling in other MGC transduction systems.

### CD-GCAP Supports Bimodal Ca^2+^ Switching of ROS-GC Activity

Discovered contemporaneously with GCAPs, retinal CD-GCAP presented itself as another Ca^2+^-dependent regulator of ROS-GC (reviewed in Sharma et al., 2014). CD-GCAP turned out to be a conformational isomer of brain S100B (the only commercially available form; Pozdnyakov et al., 1995, 1997; Duda et al., 1996a, 2002; Margulis et al., 1996; Wen et al., 2012). But in contrast to the Zn^2+^-bound brain form, the retinal form has Ca^2+^-bound. The distinction is significant because the Zn^2+^-bound form inhibits while the Ca^2+^-bound form stimulates ROS-GC1 (Pozdnyakov et al., 1997). The rest of the review will concern itself only with the retinal form.

S100B can be chemically cross-linked to the ROS-GC1 dimer giving evidence for a direct interaction. In recombinant systems, S100B binds ROS-GC1 with a K_1/2_ of 198–395 nM (Duda et al., 2002) and stimulates cyclic GMP synthesis with a K_1/2_ for Ca^2+^ of ~400 nM (Duda et al., 1996a). Deletion constructs of ROS-GC1 and peptide competition studies map the interaction to C-terminal segments: aa962–981 and aa1030–1042. Within these segments, an R^966^-IHVNS^972^ motif is obligatory for binding while a flanking cluster, R^1039^–RQK^1042^, that does not contribute to S100B binding, promotes maximal ROS-GC1 activation (Duda et al., 2002). As a diminutive protein with an estimated molecular weight of 10 kDa and only 2 EF hands, it organizes into a tetrameric subunit of the MGC complex (Donaldson et al., 1995).

**Figure 5:**
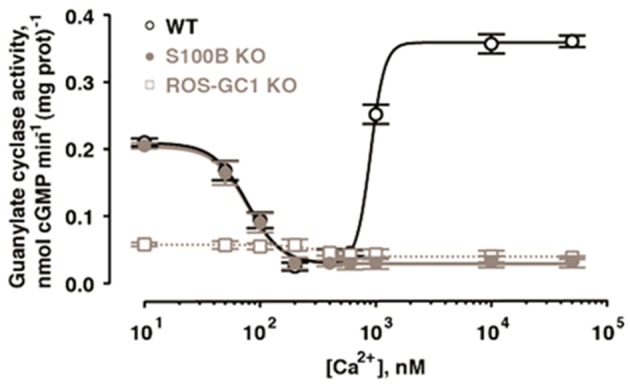
**Bimodal Ca^2+^ switching in photoreceptor to bipolar cell synaptosomal membranes of WT mice.** Stimulation of catalytic activity at high Ca^2+^ is missing from membranes of S100B^−/−^ mice and from those of ROS-GC1^−/−^ mice. Error bars show SEM. Modified with permission from Wen et al. (2012); copy right S. Karger AG., Basel.

With the ability to couple to GCAPs and to S100B, ROS-GC1 could function as a bimodal Ca^2+^ switch in cells expressing all three components. The hypothesis was explored at the photoreceptor-bipolar synaptic region of the bovine retina where prior immunohistochemical studies had demonstrated the co-presence of ROS-GC1 with GCAP1 and S100B (Liu et al., 1994; Cooper et al., 1995; Duda et al., 2002). Indeed, ROS-GC1 activity is high in synaptosomal membranes from retina at 10 nM Ca^2+^, decreases as Ca^2+^ is raised to a few hundred nM and then rises again as Ca^2+^ exceeds 1 μM (Venkataraman et al., 2003; Figure 5).

The presence of ROS-GC1, GCAPs and S100B in the outer segments of the retinal photoreceptors (Cuenca et al., 1998; Kachi et al., 1999; Rambotti et al., 1999) implicated a role for bimodal switching in phototransduction. The possibility was probed with mouse knockout models: S100B^−/−^, GCAP1^−/−^, GCAP2^−/−^, GCAP1/GCAP2^−/−^, ROS-GC1^−/−^. Biochemical experiments on murine outer segments demonstrate functional linkage of S100B with ROS-GC1 but not ROS-GC2 at [Ca^2+^]_i_ > 200 nM in the generation of cyclic GMP. Although these concentrations exceed the physiological range for Ca^2+^ in mouse ROS (Woodruff et al., 2002), they may be appropriate for cones, which sustain higher levels of Ca^2+^ in darkness. It therefore makes sense that the most recent immunocytochemistry co-localizes S100B and GCAP1 with ROS-GC1 in murine retinal cone outer segments but not ROS (Wen et al., 2012).

### Bicarbonate Controls a Ca^2+^-Independent Mode in ROS-GC that is Interlaced with Ca^2+^-Sensing Modulators

**Figure 6:**
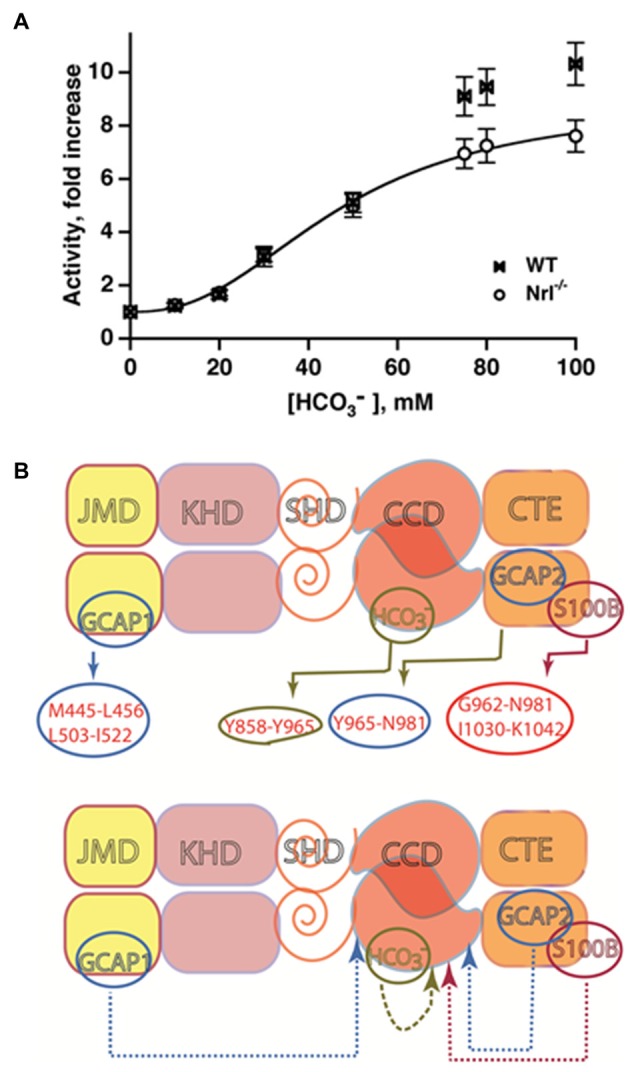
**Bicarbonate modulation of ROS-GC activity. (A)** Stimulation of ROS-GC in photoreceptor outer segment preparations from WT and neural retina leucine zipper transcription factor knockout (NRL^−/−^) mice. NRL^−/−^ photoreceptors express ROS-GC1 and GCAP1 exclusively. The dependence of guanylate cyclase activity on bicarbonate is cooperative with an EC_50_ of 47 mM. The elevated activity at high bicarbonate concentration in WT outer segments is attributed to their additional expression of GCAP2. Error bars show SEM (Duda et al., 2015). **(B)** Ca^2+^-dependent and -independent modulators of ROS-GC 1 activity. Upper panel: three Ca^2+^ sensor proteins—GCAP1, GCAP2 and S100B— and one Ca^2+^-independent modulator, bicarbonate, target individually the indicated domains within the intracellular portion of ROS-GC1. Lower panel: the targeted domains are specific switches all of which signal activation of the catalytic domain. The signaling pathways are indicated as dashed arrows.

In trailblazing studies, the MGC in olfactory neuroepithelium [olfactory neuroepithelial guanylate cyclase (ONE-GC), see below] was shown to be a sensor of atmospheric carbon dioxide (Hu et al., 2007; Guo et al., 2009). The mediator of the carbon dioxide signaling was bicarbonate. Bicarbonate did not stimulate recombinant ANF-RGC, CNP-RGC, STa-RGC, or ROS-GCs in these first studies, leading to the conclusion that among MGCs, bicarbonate was an activator unique to ONE-GC (Guo et al., 2009; Sun et al., 2009). Truncation mutants point to an interaction of bicarbonate directly with the broadly defined CCD to spur the generation of cyclic GMP (Sun et al., 2009; Duda and Sharma, 2010). The exclusive responsivity of ONE-GC to bicarbonate seemed odd because there is 85% structural conservation of its catalytic domain across MGCs. Revisiting the issue showed that the catalytic activities of recombinant bovine ROS-GCs are stimulated robustly by bicarbonate, ROS-GC1 with an ED_50_ of 27 mM and a Hill coefficient of 2.8, and ROS-GC2 with an ED_50_ of 39 mM and a Hill coefficient of 2.3 (Duda et al., 2015). The Hill coefficients >2 are consistent with one or more bicarbonate molecules binding to each monomer. Bicarbonate has a similar effect on ROS-GCs in photoreceptor outer segment membrane preparations (Figure 6A). ROS-GC’s catalytic activity remains constant over a range of pH from 7 to 9, ruling out a pH dependent mechanism for bicarbonate stimulation. The conflicting results from the first two studies may be related to the type of expression system because although bicarbonate lowers the activity of ANF-RGC expressed in CHO, the effect is not so marked with the same MGC expressed in *E. coli* or in HEK-293 (Guo et al., 2009; Sun et al., 2009).

The bicarbonate signal transduction of ROS-GC1 occurs independently of [Ca^2+^]_i_. Yet, it synergizes with the Ca^2+^-sensors: GCAP1, GCAP2 and S100B to intensify Ca^2+^ modulation, particularly for GCAP2 (Duda et al., 2015, 2016). The effect on photoreceptors is to elevate the circulating current, decrease sensitivity to flashes and accelerate flash response recovery. As a charged molecule, bicarbonate does not freely cross membranes and gains access to ROS-GC in ROS by entering through the inner segment/synapse of intact rods. In contrast, bicarbonate can access ROS-GC from the inner and outer segments of red-sensitive cones. The basis is under active investigation. These findings clarify a large body of seemingly controversial studies surrounding bicarbonate and cyclic GMP synthesis in retinal photoreceptors and provided a clue that bicarbonate signaling would be characteristic of most, if not all MGCs. A model showing the interlaced Ca^2+^-dependent and -independent pathways in the photoreceptors is depicted in Figure 6B.

An F^514^S mutation in ROS-GC1 causes Leber’s congenital amaurosis type 1 blindness in human patients (Perrault et al., 1996, 1999; Rozet et al., 2001). There is a 10-fold loss in ROS-GC1 catalytic activity (Duda et al., 1999) that is almost totally insensitive to GCAP1 modulation, despite retention of GCAP1 binding to ROS-GC (Duda et al., 2016). It follows that the loss in GCAP1 modulation occurs at the signal transduction level and possibly resides in one or more of the catalytic core residues: D^834^, E^874^, D^878^, R^925^, C^946^, N^953^. In contrast, the mutation does not abolish Ca^2+^-modulation by GCAP2 or by S100B (Duda et al., 1999, 2016), even though the absolute activities are reduced in all conditions. The interaction of this disease-causing mutation with bicarbonate led to some insights into the intramolecular signaling pathways.

Bicarbonate partially raises basal catalytic as well as GCAP2- and S100B-stimulated activities of the F^514^S mutant but does little for the deficit in GCAP1 stimulation. The restorative capacity of bicarbonate indicates that it operates downstream or independently of the F^514^S mutation. At the basic level these findings support the earlier conclusion that the S100B- and GCAP2-modulated pathways within ROS-GC1 overlap (Duda et al., 2002) but that both are distinct from the GCAP1-modulated pathway (Duda et al., 1996a, 2012c; Krishnan et al., 1998; Koch et al., 2010; Koch and Dell’Orco, 2013).

At a clinical level, high enough bicarbonate levels could provide relief for patients expressing the F^514^S-mutant ROS-GC by restoring some basal and GCAP2-modulated guanylate cyclase activity in rods and cones. Mice stricken with the mutation would not be so fortunate, since their cones express GCAP1 to the exclusion of GCAP2 (Xu et al., 2013).

### Another Ca^2+^ Sensor, Neurocalcin δ (NCδ) Is Expressed in Retinal Neurons

The failure of GCAP1 to recognize the KHD of ANF-GC and satisfy its Ca^2+^ requirement (Duda et al., 1996a) meant that other Ca^2+^-sensing subunits participate in MGC signaling. The neuronal calcium sensor (NCS) neurocalcin δ (NCδ) was tested as a potential ROS-GC regulator because it shares ~35% sequence identity with GCAPs and is expressed in the inner retina. Ca^2+^-free bovine brain NCδ has no effect on recombinant ROS-GC1, but the Ca^2+^-bound form stimulates it with a K_1/2_ for Ca^2+^ of 0.8 μM (Krishnan et al., 2004). Stimulation is ROS-GC1-specific; it does not occur with ROS-GC2. In these respects NCδ is functionally more similar to S100B than to GCAPs. NCδ bears four EF hands while S100B has only two. Despite the poor sequence identity of S100B to the C-terminal half of NCδ (GenBank accession numbers NP_001029727.1 and NP_776823.1, respectively), a comparison of their Ca^2+^-bound crystal structures reveals that the four helix-packing arrangements of their EF hands are very similar (Kumar et al., 1999). Thus, NCδ and S100B are structural and functional analogs.

NCδ purified from bovine retina exists as a homodimer of 20 kDa proteins (Krishnan et al., 2004; Venkataraman et al., 2008). Like other NCSs (Ladant, 1995; Frins et al., 1996), NCδ exhibits a Ca^2+^-dependent mobility shift. Of the four EF-hand motifs, only three—EF2, EF3, EF4—are predicted to bind Ca^2+^ (Okazaki et al., 1992; Terasawa et al., 1992; Vijay-Kumar and Kumar, 1999). The N-terminus is blocked, predominantly by myristoylation, a feature in common with all other members of the NCS-protein family except the Kv-channel interacting protein subfamily (Braunewell and Gundelfinger, 1999; Burgoyne and Weiss, 2001). Many members, NCδ included, operate as a Ca^2+^ myristoyl switch wherein the myristoyl group lies buried within a hydrophobic pocket in the absence of Ca^2+^, but is exposed when Ca^2+^ is bound to increase the membrane affinity of the protein (Lim et al., 2014). Without Ca^2+^, NCδ has no affinity for ROS-GC1. Yet from a functional standpoint, NCδ behaves as a true subunit of ROS-GC1, because the resting intracellular Ca^2+^ concentration of 100–200 nM is sufficient to keep NCδ membrane bound and associated with ROS-GC1. Stability enables a response of the MGC complex on a millisecond time scale. This concept has been experimentally validated. NCδ and ROS-GC1 are present together in the IPL under resting conditions and the addition of Ca^2+^ stimulates native ROS-GC1 activity (Krishnan et al., 2004); the myristoyl group of NCδ causes a 2-fold amplification of the saturation activity of ROS-GC1. The binding kinetic parameters of the Ca^2+^-bound NCδ with ROS-GC1 are (Krishnan et al., 2004): *K*_A_ = 2.3 × 10^6^ M^−1^ and *K*_D_ = 4.6 × 10^−7^ M.

Mapping studies place binding of NCδ at aa732–962, a site that does not overlap with GCAP1-, GCAP2-, or S100B-modulated domains of ROS-GC1 (Krishnan et al., 2004). Finer analyses pinpoint the NCδ-modulated site between V^836^-L^857^ of ROS-GC1 (Note: the sequence numbering in the following reference is offset by one amino acid; Venkataraman et al., 2008), within the CCD. This segment is accessible to the solvent and fits comfortably in the V-shaped crevice of NCδ, bounded by the EF-1 hand residues. These residues form a special hydrophobic–hydrophilic patch, which may be a distinctive feature of the Ca^2+^-dependent signaling property of NCδ. It has a secondary structure of helix-loop-helix and is acidic in nature with a pI of 3.37.

Traditionally, the belief had been that the CCD, located at the C-terminus of the protein, was isolated from all of its ligands and depend on upstream modules to translate the ligand signals for its activation to generate cyclic GMP. The localization of NCδ binding, which predated work on the bicarbonate binding site, along with new insights on the antiparallel nature of the catalytic center (Duda et al., 2012c), called for a new signaling model in which the isolated core catalytic module by itself is dimeric in nature with intrinsic basal guanylate cyclase activity. The two chains of the catalytic module are antiparallel. Ca^2+^-bound NCδ interacts directly with the ROS-GC1 core catalytic site without need for the adjacent N-terminally located α-helical “dimerization domain” structural element (L^770^-P^807^). Because this dimerization domain plays a role in transmitting the signal from GCAP1 to the catalytic module (Zägel et al., 2013), it is now referred to as signaling helix domain (SHD).

ROS-GC1 couples with another NCS in the inner retina, most likely a GCAP, because cyclic GMP synthesis increases at very low Ca^2+^ (Krishnan et al., 2004). If the entire system is present in the same cells, then it could function as bimodal switch like that at the photoreceptor-bipolar cell synapse, except that NCδ replaces S100B.

### NCδ Is a Ca^**2+**^ Sensor Modulating ANF-RGC

Based on the rationale that the binding site for NCδ lies in the catalytic center of ROS-GC, a domain that is conserved in all MGCs, NCδ was a prime candidate to be the Ca^2+^ sensor for ANF-RGC. The V^836^-L^857^ site in ROS-GC1 corresponds with the V^851^-L^872^ site of ANF-RGC with a sequence identity of 68.2%.

The first step was to test myristoylated NCδ against recombinant ANF-RGC. NCδ stimulates the catalytic activity of ANF-RGC in a dose-dependent fashion with an EC_50_ of 0.5 μM Ca^2+^ (Duda et al., 2012b). As is the case with ROS-GC1 and GCAPs, ATP/ANF is not needed for stimulation, further demonstrating that the Ca^2+^-modulated modes are distinct from that of hormonal, ANF signal transduction. These properties broke down the traditional concept that ANF-RGC was strictly a transducer of the natriuretic peptide hormone ANF. A second, Ca^2+^-modulated pathway regulates the same physiological processes of natriuresis, diuresis and blood pressure.

Only myristoylated NCδ stimulates the Ca^2+^-modulated ANF-RGC catalytic activity, the non-myristoylated form is ineffectual. It is now appreciated that in a previous study, Ca^2+^/NCδ did not stimulate ANF-GC (Kumar et al., 1999) because it was unmyristoylated, underscoring the importance of this posttranslational modification. Myristoylated NCδ controls the activity of ANF-RGC by lowering its K_m_ for GTP substrate and by increasing its catalytic efficiency, *k*_cat_, from 6.5 ± 0.3 to 41.4 ± 0.5 pmol cyclic GMP/s. The functional unit of the Ca^2+^-bound complex is composed of NCδ dimer and ANF-RGC dimer.

To assess an overlap between the Ca^2+^- and the ANF-modulated pathways, ANF-RGC catalytic activity was analyzed first in the presence of 1 μM Ca^2+^ and 2 μM myristoylated NCδ and then with increasing concentrations of ANF, ranging from 10^−11^ to 10^−6^ M, and constant 0.8 mM ATP. The responses were enhanced 3.5- and 4.5-fold alone, and about 15-fold in combination. Thus the Ca^2+^-modulated and the hormone-modulated pathways can operate independently but when both are in play, the multiplicative power suggests a convergence in their control of ANF-RGC transduction activity.

To link cardiovascular biochemistry with physiology, a hemizygous NCδ knockout mouse, NCδ^+/−^, was generated (Duda et al., 2012b). We were unsuccessful in generating viable homozygous NCδ knockout mice. The adrenocortical zona glomerulusa was chosen as the subject of investigation because the ANF-RGC signal transduction system is present in glomerulosa cells (Takayanagi et al., 1987; Sharma et al., 1989b; Duda et al., 1991; Rondeau et al., 1995), and NCδ is expressed in the same layer (Nakano et al., 1993). The particulate fraction of the adrenal gland from NCδ^+/−^ mice yields 50% of the normal ANF-RGC activity and addition of saturating amounts of exogenous NCδ and Ca^2+^ reconstitutes the full MGCs activity. Thus, the Ca^2+^-dependent NCδ-modulated ANF-RGC signaling pathway exists in the adrenal glands of the mice and, as anticipated, it is only functionally half as active in NCδ^+/−^ mice compared to wild type mice.

ANF-RGC catalytic activity in the adrenal gland offsets the renin-angiotensin aldosterone system; it inhibits aldosterone synthesis and thereby lowers blood pressure (Burnett et al., 1984; Aoki et al., 2000; Shi et al., 2001). Aldosterone levels in the plasma of NCδ^+/−^ mice are 27% higher over the control NCδ^+/+^ mice (Duda et al., 2014). Together with the evidence that corticosterone levels synthesized by fasciculata cells are unaffected by the absence of NCδ gene and immunohistochemical studies showing that NCδ and ANF-RGC are co-expressed in the glomerulosa cells of the mouse adrenal gland, the conclusion is that the Ca^2+^-modulated ANF-RGC transduction machinery resides specifically in aldosterone producing glomerulosa cells of the adrenal gland (Duda et al., 2012b). Importantly, it is physiologically linked with blood pressure regulation. Systolic blood pressure of the NCδ^+/−^ mice is 38% higher than in wild type mice, 127 vs. 92 mm Hg.

**Figure 7:**
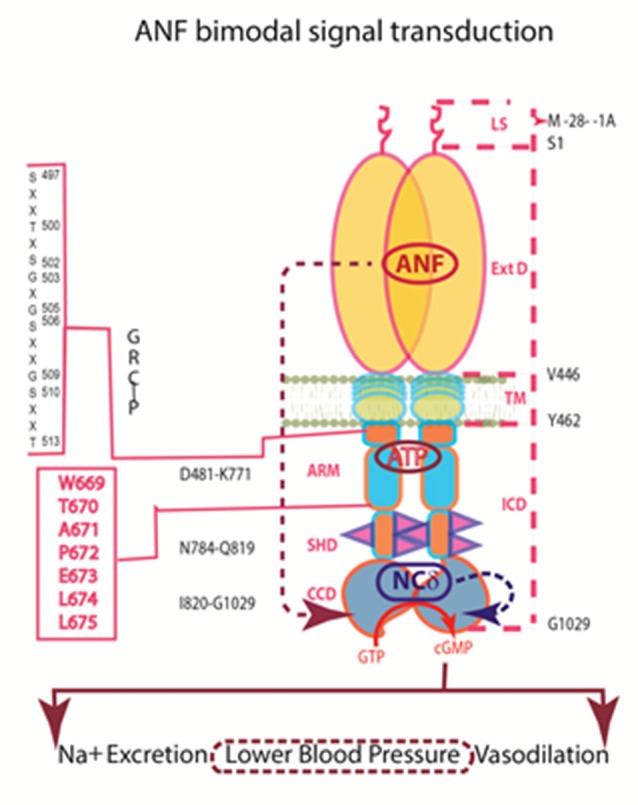
**Independence of the signaling pathways of ANF/ATP and of NCδ.** The trajectory of the ANF pathway (maroon dashed arrow) originates at the ExtD and passes through the TM, ARM and signaling helix domain (SHD) in its course to CCD. In contrast, the trajectory of the NCδ pathway (blue dashed arrow) lies within the CCD. The ANF-RGC dimer is thought to bind a dimer of NCδ, but only a single subunit is shown. Both pathways are the physiological regulators of the mouse blood pressure.

In conclusion, ANF-RGC performs two transductions in some systems. One signal arrives externally in the form of potent hormones, ANF and brain natriuretic peptide (BNP), which bind to an external surface domain. The second signal is internal; a neurocalcin subunit monitors intracellular Ca^2+^ and directly modulates the catalytic domain (Figure 7). This Ca^2+^-modulation defines a new paradigm of ANF-RGC signaling and unveils an alternate pathway to the physiological control of the endocrine systems that avert hypertension. The NCS VILIP-1 can bind to ANF-RGC (Braunewell et al., 2001). If VILIP-1 modulates activity of ANF-RGC as it does for CNP-RGC, it may substitute for NCδ in other systems.

### An MGC (ONE-GC, GC-D) Detects Odorants

Cloning of a MGC, termed GC-D, from a total olfactory cDNA library (Fulle et al., 1995) came at a time when olfaction was thought to occur solely through G protein coupled receptors and the cyclic AMP signaling pathway (Buck, 1995; Belluscio et al., 1998; Breer, 2003; Lai et al., 2005). Even though an odorant for GC-D was not yet known, there were clues for a cyclic GMP transduction system in olfaction. *In* *situ* hybridization and immunocytochemical studies indicate that GC-D co-expresses with a cyclic GMP stimulated phosphodiesterase, PDE2, that hydrolyzes cyclic GMP as well as cyclic AMP and an ion channel that is gated specifically by cyclic GMP (Fulle et al., 1995; Juilfs et al., 1997; Meyer et al., 2000). GC-D/PDE2 containing neurons constitute only a small subpopulation of neuroepithelial neurons for which components of the “standard” olfactory cyclic AMP signaling system: G_olf_, ACIII, PDE1C2, and the α3 and β1b subunits of the cyclic nucleotide gated ion channel, are missing. Moreover, they project to specific “necklace glomeruli” of the olfactory bulb. Interestingly, immunoprobes with different anti-GC-D antibodies uncover more extensive labeling of olfactory neuroepithelial cilia suggesting the possible expression of more than one type of MGC and/or a subsidiary role for cGMP in a majority of the olfactory receptors (Juilfs et al., 1997; Duda et al., 2001a).

A Ca^2+^-modulated ROS-GC1 system is absent from rat olfactory neuroepithelium and its ciliary subcompartment where odorants are transduced, so to assess the possibility that a novel MGC is involved, the authors cloned ONE-GC directly from a rat olfactory neuroepithelium cDNA library (Duda et al., 2001a). Sequence alignment analysis disclosed that ONE-GC has complete primary structural identity with the prior cloned GC-D (Juilfs et al., 1997), yet only partial 47.9% and 47.6% identity with ROS-GC1 and ROS-GC2, respectively. A polyclonal antibody raised against its unique 12 amino acid C-terminal epitope revealed that it shares no immunological identity with ROS-GC1 nor with ROS-GC2 (Duda et al., 2001a). Thus, ONE-GC and GC-D are the same guanylate cyclase. Because ONE-GC resides in the olfactory neuroepithelium compartment, the authors prefer the ONE-GC nomenclature over GC-D.

The Ca^2+^-modulated myr-NCδ-ONE-GC system meets five criteria set forth to secure its role as a genuine, Ca^2+^-modulated odorant transducer (Duda et al., 2001a, 2004; Duda and Sharma, 2008; Sharma and Duda, 2010): (1) ONE-GC responds to odorant in its native state. Uroguanylin is an extremely effective stimulus for ONE-GC expressing neurons (Leinders-Zufall et al., 2007), binding the ExtD of ONE-GC with an EC_50_ of 20 pM and saturating it at 500 pM (Duda and Sharma, 2008). Interestingly, STa-RGC binds uroguanylin and guanylin, but ONE-GC is more selective and does not recognize guanylin; (2) The response to odorant is relatively fast for an amplifying system; (3) ONE-GC resides within the membrane portion of the cilia; (4) It is modulated by physiological concentrations of free Ca^2+^ with a *K*_1/2_ of 700 nM, generating responses similar to that of odorant; and (5) The Ca^2+^-responsive system can be reconstituted with recombinant myr-NCδ and ONE-GC.

NCδ binds M^880^-L^921^ of ONE-GC, a segment encompassing the corresponding V^836^-L^857^ binding site in ROS-GC1 (see above), affording direct access to the catalytic domain (Duda and Sharma, 2008). The key ^844^MSEPIE^849^ motif in ROS-GC1 is present in ONE-GC as ^908^LSEPIE^913^. *In vivo* cell studies demonstrate that in resting cells, the NCδ-ONE-GC complex is exclusively at the plasma membrane region (Duda et al., 2004). Assessed by the SPR spectroscopy, kinetic parameters of the Ca^2+^-bound NCδ binding to ONE-GC are: *K*_D_ = 2.8 × 10^−7^ M; *k*_on_ = 5.7 × 10^3^ M^−1^s^−1^; *k*_off_ = 1.56 × 10^−3^ s^−1^.

**Figure 8:**
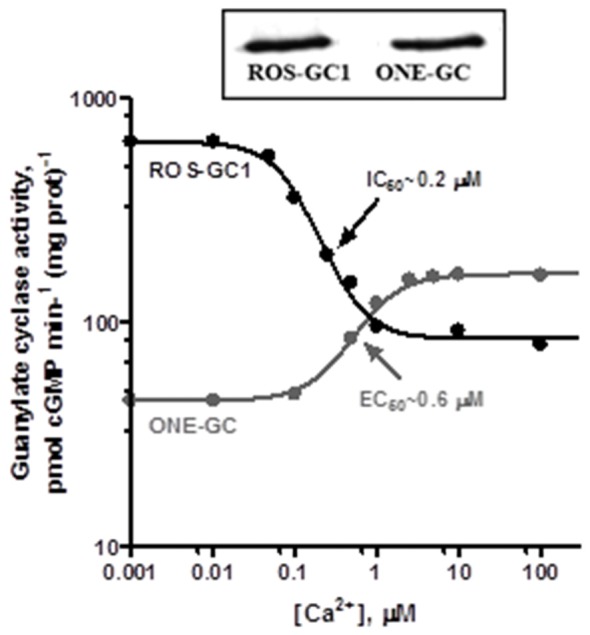
**Antithetical Ca^2+^ modulation of ROS-GC1 and olfactory neuroepithelial guanylate cyclase (ONE-GC) activities by GCAP1.** In the presence of GCAP1, the catalytic activity of recombinant ROS-GC1 decreases as Ca^2+^ is raised from 1 nM to 100 μM, but the catalytic activity of recombinant ONE-GC increases. Western blots confirming ROS-GC1 and ONE-GC expression are shown above. Redrawn from Duda et al. (2012a).

Gene-knockout studies in mouse establish that in olfactory neuroepithelium another NCS, hippocalcin (Hpca; Krishnan et al., 2009) activates ONE-GC catalytic activity in the Ca^2+^ K_1/2_ range of 0.5–0.7 μM. Besides neurocalcin and Hpca, ONE-GC interacts with yet one more Ca^2+^ sensor, GCAP1 (Pertzev et al., 2010). The interaction was missed initially, because it is antithetical to that with ROS-GC in phototransduction. Instead of stimulating ONE-GC at the low nM range of free Ca^2+^ (i.e., in the Ca^2+^ free state), GCAP1 stimulates at the upper range of Ca^2+^ (i.e., in the Ca^2+^ bound state; Duda et al., 2006, 2012a; Figure 8). It was the first, and is to date, the only example of an NCS switching the directionality of its action with a change in binding partners. To add to the intrigue, stimulation of ONE-GC activity by GCAP1 occurs at an EC_50_ that is higher than the apparent IC_50_ for ROS-GC1 activity. Biochemical assays with antibodies indicate that 35% of the total ONE-GC transduction activity is controlled by GCAP1, 27% by NCδ, and 38% by Hpca (Krishnan et al., 2009; reviewed in Sharma and Duda, 2010).

ONE-GC resembles the ANF-RGC in combining a surface hormone/odorant transduction with internal Ca^2+^ sensing. Yet, a primary sequence identity of only 28.1% with ANF-RGC but a sequence identity of 47.6% with ROS-GC makes it closer akin to the latter subfamily. Like photoreceptor ROS-GCs, ONE-GC is stimulated by bicarbonate (Guo et al., 2009; Sun et al., 2009). The target site of the bicarbonate signal resides within Y^922^-P^1028^ segment of the catalytic domain (Hu et al., 2007; Sun et al., 2009; Duda and Sharma, 2010).

### Additional Ca^2+^ Sensors Couple to MGC in Hippocampus

The Ca^2+^ sensor frequenin (Frq, also termed NCS type-1, NCS-1) is the most primordial member of the NCS protein family with homologs already present in yeast (Fik-Rymarkiewicz et al., 2006; reviewed in Sharma and Duda, 2012). While frequenin supports Ca^2+^ modulation with numerous targets, its status with respect to MGCs was unknown. It was of interest to test MGCs because the results could provide clues about the timeline for the evolutionary appearance of NCS-MGC interactions. Bovine Frq has a subunit mass of 22 kDa and shows a Ca^2+^-dependent gel mobility shift, a characteristic of the NCS family (Ladant, 1995). Its structure is highly conserved on the evolutionary ladder: 100% identity with chicken (Nef et al., 1995; Olafsson et al., 1997; McFerran et al., 1999) rat, and human (Jeromin et al., 1999; Bourne et al., 2001); 98% with frog (Olafsson et al., 1995), 75% with *C. elegans* (De Castro et al., 1995), 72% with *Drosophila* (Pongs et al., 1993), and 60% with the yeast form (Hendricks et al., 1999). In common with the NCS family trait, there are four EF-hands, yet only three—2, 3 and 4—are functional in binding Ca^2+^. Frq is myristoylated but its operation as a Ca^2+^ myristoyl switch is somewhat controversial (Lim et al., 2011).

Co-immunoprecipitation experiments demonstrate that Frq and a ONE-GC-like MGC exist as a complex in bovine hippocampal neurons (Fik-Rymarkiewicz et al., 2006). When these studies were conducted, no Ca^2+^-modulated MGC transduction machinery had ever been reported in hippocampus. The existence of the complex is Ca^2+^-dependent, dissociating when the Ca^2+^ is depleted with EGTA. Interestingly, a small fraction of Frq immunoprecipitates with the hippocampal MGC even in the presence of 5 mM EGTA. The MGC and the native NCS from hippocampal neurons respond to [Ca^2+^] with a five-fold increase in activity and a K_1/2_ for Ca^2+^ of 0.7 μM. The EC_50_ of hippocampal MGC for recombinant myr-Frq is 0.7 μM, and at saturating levels of Ca^2+^, activity increases five-fold. Recombinant myr-Frq robustly stimulates the catalytic activity of recombinant ONE-GC 6-fold with an EC_50_ of 0.7 μM, only marginally (~1.5-fold) stimulates recombinant ROS-GC1, and utterly fails to stimulate ROS-GC2. The binding kinetics between Frq and the M^836^-C^1110^ segment of ONE-GC were determined through surface plasmon resonance spectroscopy: K_D_ =0.43 μM; *k*_on_ (the association rate constant) = 7.14 × 10^3^M^−1^s^−1^; *k*_off_ (dissociation rate constant) = 3.04 × 10^−3^s^−1^; and *K*_A_ (equilibrium association constant) = 2.35 × 10^6^ M^−1^. Frq therefore may be a soluble modulator rather than a true subunit of the hippocampal MGC. Functionally, a mixed distribution of Frq bound to a minor fraction of the hippocampal MGC and free Frq in the cytoplasm of resting neurons could serve to effectively raise the Ca^2+^ requirement, extend the Ca^2+^ range and lengthen the duration of the rise in Ca^2+^ necessary for stimulation of cyclic GMP synthesis. Modulation of an MGC by myr-Frq suggests that during evolution, Ca^2+^-sensors that stimulate MGCs at high Ca^2+^ probably arose before the GCAPs that stimulate at low Ca^2+^.

These results argue in favor of a ONE-GC-like MGC in hippocampus, but its identity remains elusive because GC-D is not expressed in bovine. Hippocalcin, which can couple with ONE-GC (*vide supra*, Krishnan et al., 2009), is potentially a second soluble Ca^2+^ modulator of MGCs in hippocampus. CNP-RGC is another hippocampal MGC, responding to CNP produced locally (Langub et al., 1995; Herman et al., 1996). Mutant rats with a defective response to CNP show disturbances in long term potentiation and long term depression (Barmashenko et al., 2014). These studies on hippocampus begin to unravel a role for MGCs in learning and memory.

### A Novel MGC (GC-G) Responds to Mild Cooling

**Figure 9:**
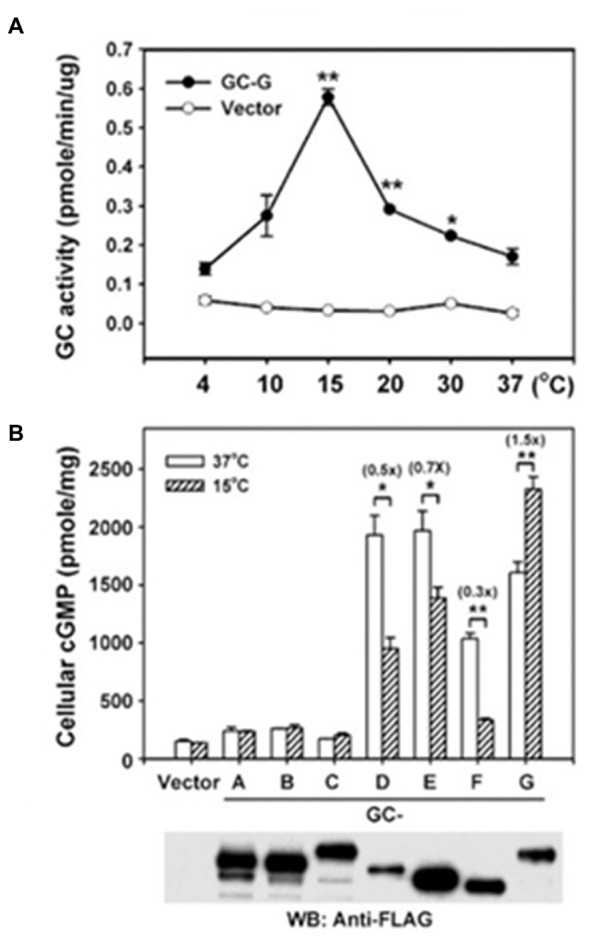
**Thermosensing by GC-G. (A)** Temperature dependence of GC-G activity. Membranes of HEK293T cells transfected with GC-G cDNA or empty vector were used to determine the guanylate cyclase activity at the indicated temperatures for 20 min. **(B)** Uniqueness of GC-G in its stimulation by cool temperature. Expression plasmids encoding FLAG-tagged versions of all known MGCs were transfected into HEK-293T cells and their membranes were used for guanylate cyclase activity assays at 37 and 15°C. ANF-RGC, CNP-RGC, and STa-RGC showed little change, whereas ROS-GC1, ROS-GC2 and ONE-GC showed greater activity at 37°C. Error bars show SD. ^*^p < 0.05, ^**^p < 0.01. Analysis by Western blot with anti-FLAG antibodies confirms the expression of each MGC. Reproduced with permission from Chao et al. (2015): Receptor guanylyl cyclase-G is a novel thermosensory protein activated by cool temperatures. EMBO J 34:294-306, © John Wiley and Sons, Inc.

A seventh MGC, GC-G, was cloned from rat with some suggestion for alternative splicing (Schulz et al., 1998). The realization that GC-G is expressed in selected, temperature- sensitive neurons of the Grueneberg Ganglion in the nasal cavity of mouse (Fleischer et al., 2009) led to the remarkable discovery that GC-G is a thermosensor (Chao et al., 2015), reminiscent of MGCs encoded by gcy-8, gcy-18 and gcy-23 in *C. elegans* (Takeishi et al., 2016). The molecular mechanism has not yet been defined but it would appear that dimerization of the CCD is optimal near 15°C with a sharp temperature dependence (Figure 9). GC-G is multifunctional—it also responds to the predator odorant 2,4,5-trimethylthiazoline (Chao et al., 2015) and to bicarbonate (Chao et al., 2010). Modulation by Ca^2+^ is not yet known. Activation of Grueneberg Ganglion neurons by mild cooling elicits ultrasound vocalization in pups, a behavior that is attenuated in GC-G knockouts (Chao et al., 2015).

### New Functions of Ca^2+^-Interlocked MGCs Continue to Be Uncovered

GC-D has degraded to a pseudogene in most primates (Young et al., 2007), and GC-G appears to have lost its capacity to function as a cyclase in humans (Yang et al., 2010), nevertheless, there is growing appreciation for the extensive roles that the other MGCs have taken on throughout the body (Kuhn, 2016). A few examples that we have begun to explore are listed below.

In the pineal gland, adrenergic receptor activity is coupled to cyclic GMP synthesis by ROS-GC1 and changes in intracellular Ca^2+^ via S100B (Venkataraman et al., 1998). GCAP1 expression in the pineal gland raised the possibility for another bimodal switch like that present in photoreceptor-bipolar cell synapses (Venkataraman et al., 2000). However, immunohistochemical analyses distinguish two types of pinealocytes, one that is GCAP1-modulated and another that is S100B-modulated. Therefore, bimodal switching is precluded in this system. ANF, CNP and CNP-RGC are also expressed in pinealocytes (Olcese et al., 1994; Middendorff et al., 1996).

Expression of a Ca^2+^ signaling MGC transduction system in the anterior portion of the bovine gustatory epithelium has been demonstrated at the biochemical, molecular and functional levels, implicating a role in taste (Duda and Sharma, 2004). The system is composed of two components: the Ca^2+^-sensor protein, S100B and the transducer, ROS-GC1. Co-immunoprecipitation experiments reveal that ROS-GC1 and S100B physically interact with each other. The precise operational mechanism of this signal transduction mechanism has not yet been decoded at the physiological level.

Dissection and reconstitution of the biochemical and functional components of the rat olfactory bulb demonstrate that a GCAP1-modulated ROS-GC1 transduction system is present in its mitral cells. At the biochemical level, the machinery functions according to the principles established for phototransduction. Olfactory bulb ROS-GC1 senses free levels of Ca^2+^ via GCAP1 with an IC_50_ of 70 nM. The bulb neurons do not express GCAP2 Ca^2+^ sensor, nor do they express ROS-GC2. The olfactory bulb functions as a “processing unit” for post-odorant transduction events, directing the odorant impulses to the olfactory tract on their way to cortical centers of the brain for odorant perception (Figure 6; Duda et al., 2001b).

Numerous studies support second messenger roles for GMP and for the presence of an ANF-dependent ANF-RGC transduction system in the physiology of the testes: sperm motility, development of testicular germ cells, relaxation of peritubular lamina propia cells, testosterone synthesis in Leydig cells and dilation of testicular blood vessels (Marala and Sharma, 1988; Singh et al., 1988; Garbers, 1989; Pandey and Singh, 1990; Armstrong et al., 1994; Middendorff et al., 1997, 2000; Pandey et al., 1999; Mourdjeva et al., 2001).

Biochemical studies demonstrate a ROS-GC1 transduction system modulated by GCAP1 and S100B in plasma membrane of bovine testes (Jankowska et al., 2007, 2014). Immuno-stainings establish that these components co-localize in bovine and human sperm (Jankowska and Warchol, 2010). A convergence of molecular genetics, biochemistry and immunohistochemistry disclose NCδ in male gonads (Jankowska et al., 2014). Thus, three sensors: GCAP1, S100B and NCδ, capture and transmit Ca^2+^ signals to ROS-GC1 in this tissue. The transduction unit is expressed in all germinal cell types of the human testes: spermatogonias, spermatocytes, and spermatids. The spermatids residing close to the seminiferous tubule lumen embody the abundance of the transduction unit, suggesting involvement with the processes of spermatogenesis. Based on mouse-KO models, NCδ is the major (75%) and S100B the minor (25%) contributor of Ca^2+^ signaling by ROS-GC1. The contribution of GCAP1 has not been determined but there is evidence for bimodal Ca^2+^ operation in bovine and human spermatozoa (Jankowska and Warchol, 2010). Guanylate cyclase activity is minimal at 800 nM, but increases with a decline in Ca^2+^ with an IC_50_ of 200 nM and increases as Ca^2+^ is raised with an EC_50_ of 2 μM. GC-G expression in mouse sperm (Kuhn et al., 2004; Huang et al., 2006) could support thermotaxis and/or chemotaxis and contribute to the bicarbonate sensitivity. The homologous human gene is also expressed in sperm, yet it does not encode a true MGC because of termination codons in the KHD.

## Conclusions

**Figure 10:**
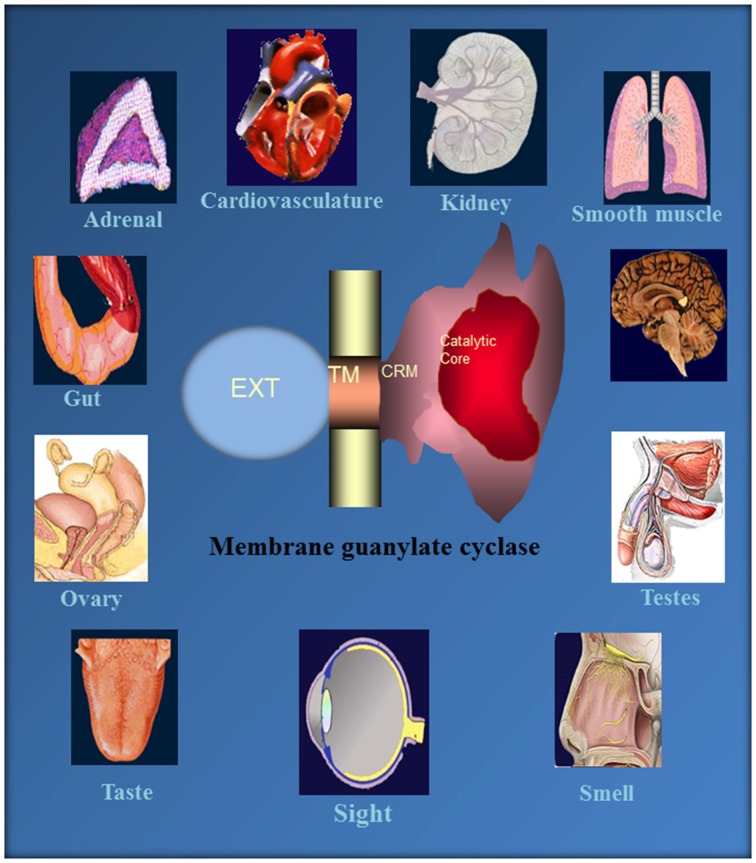
**Ubiquitous expression of MGC signaling systems.** MGCs is a multi-switching cyclic GMP generating machine linked with the physiology of cardio-vasculature, smooth muscle relaxation, sensory transduction, neuronal plasticity and memory in mammalian neurosensory, endocrine and peripheral tissues. Reproduced from Sharma et al. (2015).

This monograph narrates an extraordinary tale of MGCs. It started about four decades ago, when the authors’ group made a key observation that a novel, epinephrine-sensitive MGC transduction system existed in rat adrenocortical carcinoma 494 cells that is absent from the normal adrenal cortex (Perchellet and Sharma, 1980; Shanker and Sharma, 1980). Over the ensuing years meticulous dissection of the MGC system resulted in its rise from anonymity to its present status as a preeminent cellular signal transducer generating the intracellular second messenger, cyclic GMP, to critically control countless physiological processes, including cardiac vascular tension, smooth muscle relaxation, blood volume, cellular growth, sensory transduction, neural plasticity, learning and memory (Figure 10).

**Figure 11:**
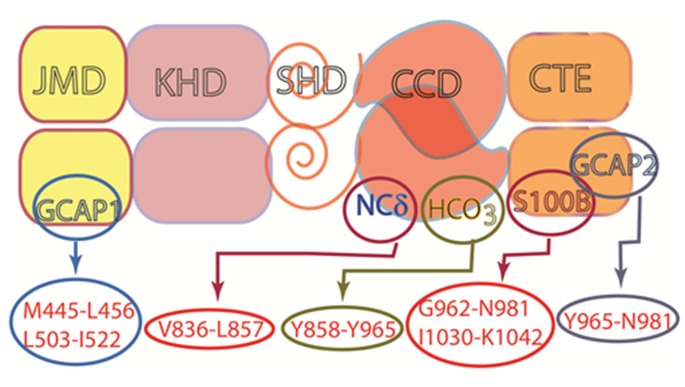
**Multiple modulators of ROS-GC1 catalytic activity.** ROS-GC is a multi-switch transduction unit with a site for HCO_3_^−^ and Ca^2+^ sensor switching domains for GCAP1 and 2, S100B and NCδ. The sites for Frq and Hpca binding are not known, but based on studies with ONE-GC, the site for Frq is likely to be located in the CCD. This architecture provides it with cellular signaling specificity and elasticity in a variety of tissues.

How does it perform such diverse tasks? A modular structure makes the MGC extremely flexible and multimodal. Unlike the three-component design of its predecessor second messenger systems: adenylate cyclase and IP_3_, G-protein and G-protein coupled receptors; the MGC transduction system consists of a single entity, a trans-membrane-spanning protein that serves as both a receptor and a signal transducer. For all MGCs except for ROS-GC1 and 2, the ExtD binds hormones or small molecules. Some MGCs have subunits that attach to an intracellular domain (ICD) and confer Ca^2+^ sensitivity. The relation between Ca^2+^ and MGC activity is variable. With ANF-RGC, high Ca^2+^ stimulates activity without ligand binding whereas ROS-GC1 and ONE-GC can act as bimodal Ca^2+^ switches; activity is maximal as Ca^2+^ is depleted, it subsides at midrange [Ca^2+^]_i_, then rises once again as [Ca^2+^] reaches its highest levels. The basis for these different modes of action trace to the identity of the Ca^2+^ sensor involved: GCAPs, S100B, neurocalcin, frequenin. In a startling arrangement, the conduct of GCAP1 depends upon its MGC partner; it stimulates ROS-GCs at low Ca^2+^ and inhibits them at high Ca^2+^, but stimulates ONE-GC at high Ca^2+^. ROS-GC1 is capable of coupling with many different Ca^2+^ sensors (Figure 11), in part because of its CTE. In contrast, ROS-GC2 is quite selective even though it also has a CTE. Interestingly, the expansiveness of CTE in STa-RGC and in GC-G raises the question as to what other functions this structure performs. It is now recognized that the conserved CCD dimer is receptive to signals generated by both C-terminal and N-terminal modular blocks. In an extraordinary development, the CCD is also subject to direct tuning by Ca^2+^/neurocalcin and by bicarbonate. With conservation of the CCD, nearly every MGC responds to bicarbonate. The response of ROS-GC to bicarbonate and Ca^2+^-modulated GCAPs and S100B in combination exceeds the sum of the individual modulations affording a means to amplify the transductions. It remains to be seen whether bicarbonate can also amplify transduction of orthosteric ligand binding. Chemical modulation and regulation by phosphorylation of MGCs are summarized in Table 1. Besides sensing odorants and bicarbonate and Ca^2+^, GC-G has evolved a remarkable mechanism whereby dimerization and catalytic activity are maximal at a particular temperature in order to sense mild cooling. These transductions encroach on territories of GPCRs and TRP channels. In the former case, MGCs may provide for faster signaling and in the latter, case, amplification. The growing appreciation for MGC signaling throughout the body calls for reclassification of their Ca^2+^ sensors. NCδ and GCAPs can no longer be considered as strictly NCSs, as their expression is more widespread.

**Table 1:** **Modulators of membrane guanylate cyclase (MGC) activity**.

Receptor		Ligands: Orthosteric, Allosteric			℗	Ca^2+^ Sensor						
Type	Alias	Agonists	ATP	${\text{HCO}}_{\text{3}}^{-}$		GCAP1	GCAP2	S100B	NCδ	Frq	Hpca	VILIP-1
ANF-RGC	GC-A	ANF, BNP	**+**	**-**?	**+**	x			✓			
CNP-RGC	GC-B	CNP	**+**	x	**+**							✓
STa-RGC	GC-C	STa, guanylin, uroguanylin	**+**	x	**-**
ONE-GC	GC-D	uroguanylin		**+**		✓			✓	✓	✓	
ROS-GC1	GC-E	orphan	**+**	**+**	**+**	✓	✓	✓	✓	✓	✓	
ROS-GC2	GC-F	orphan		**+**		x	✓	x	x	x	x	
GC-G		TMT, DMP?		**+**

*Abbreviations for ligands: ANF, atrial natriuretic factor; BNP, brain natriuretic peptide; CNP, C-type natriuretic peptide; STa, heat stable bacterial enterotoxin; TMT, 2,4,5-trimethylthiazoline; DMP, 2,3-dimethylpyrazine. The evidence supporting DMP as an agonist for GC-G is indirect. ⅷ refers to phosphorylation of Ser and Thr residues, except for GC-C, where Tyr phosphorylation reduces STa-induced activity. For the effect of ATP, ${\text{HCO}}_{\text{3}}^{-}$ and ⅷ, “+” indicates an increase in activity or a requirement for orthosteric agonist-induced activity, “−” indicates a decrease in activity. Bicarbonate decreases GC-A activity when expressed in CHO but not when expressed in E. coli or in HEK-293T. A “✓” means that the Ca^2+^ sensor has an effect on the activity of the MGC; an “x” means that it has no effect. In general, Ca^2+^ sensors stimulate MGCs when Ca^2+^ is bound. The notable exception is that for ROS-GCs, GCAPs stimulate at low Ca^2+^ and inhibit at high Ca^2+^. In some cases, the interactions were tested in a recombinant system so the coupling may not exist in vivo*.

## Nomenclature

The reasoning for usage of the present nomenclature for the subtypes of MGCs was explained in our previous review (Sharma, 2010): “From its inception to its first discovery this enzyme has been termed ‘guanylate cyclase’… For unknown reasons some investigators, subsequently, have changed its name to ‘guanylyl cyclase’. The guanylate cyclases ANF-RGC, CNP-RGC and STa-RGC have been alternately named as GC-A, GC-B and GC-C, respectively; and ONE-GC, ROS-GC1 and ROS-GC2 as GC-D, GC-E and GC-F, respectively. This investigator has preferred the former functional nomenclature of the guanylate cyclases.”

## Author Contributions

All authors contributed in the organization, design and writing.

## Conflict of Interest Statement

The authors declare that the research was conducted in the absence of any commercial or financial relationships that could be construed as a potential conflict of interest.
